# Burden and predictors of diabetic kidney disease in an adult Ugandan population with new-onset diabetes

**DOI:** 10.1186/s13104-023-06500-1

**Published:** 2023-09-28

**Authors:** Davis Kibirige, Isaac Sekitoleko, William Lumu

**Affiliations:** 1 Non-Communicable Diseases Program, Medical Research Council, Research Unit, Virus Research Institute and London School of Hygiene and Tropical Medicine Uganda, Entebbe, Uganda; 2 Department of Medicine, Uganda Martyrs Hospital Lubaga, Kampala, Uganda; 3 Department of Medicine, Mengo Hospital, Kampala, Uganda

**Keywords:** Diabetic kidney disease, Adult patients, New-onset diabetes, Uganda, Sub-saharan Africa

## Abstract

**Background:**

Despite the growing evidence of diabetic kidney disease (DKD) in adult patients with long-standing diabetes in sub-Saharan Africa, data on its burden and correlates in adult African patients with new-onset diabetes are limited. We, therefore, undertook this study to determine the burden and predictors of DKD in an adult population with new-onset diabetes in Uganda.

**Methods:**

We collected data on the relevant sociodemographic, clinical, anthropometric, and metabolic characteristics in 519 participants with newly diagnosed diabetes recruited from seven tertiary hospitals. A spot mid-stream urine sample was collected for determination of the urine albumin creatinine ratio (UACR) using Clinitek® microalbumin strips and a point-of-care Clinitek® status analyser. The estimated glomerular filtration rate (e-GFR) was determined using the Chronic Kidney Disease Epidemiology formula. The presence of DKD was defined as a spot UACR ≥ 3 mg/mmol with or without an e-GFR < 60 ml/min/1.73m^2^.

**Results:**

The median (IQR) age, UACR, and e-GFR of the participants were 48 years (39–57), 2.27 mg/mmol (1.14–3.41), and 121.8 ml/min/1.73m^2^ (105.4-133.9). UACR ≥ 3 mg/mmol and e-GFR < 60 ml/min/1.73m^2^ was noted in 175 (33.7%) and 7 (1.4%) participants, respectively. DKD was documented in 175 participants (33.7%). Compared with those without DKD, participants with DKD were more likely to be ≥ 50 years of age (53.7% vs. 43%, p = 0.02) and to have co-existing hypertension at the time of diagnosis (40.6% vs. 30.1%, p = 0.02). On multivariate analysis, self-reported hypertension comorbidity (OR 1.76 95% CI 1.24–2.48, p = 0.002) and body mass index (BMI) ≥ 30 kg/m^2^ (OR 0.61 95% CI 0.41–0.91, p = 0.02) were noted to independently predict DKD.

**Conclusion:**

In this study population, DKD was relatively common and was independently associated with self-reported hypertension comorbidity and BMI ≥ 30 kg/m^2^.

## Introduction

Diabetic kidney disease (DKD) is a heterogeneous clinical condition characterised by the presence of persistent overt proteinuria (urine albumin creatinine ratio or UACR ≥ 300 mg/g or 3 mg/mmol) and declining renal function reflected by an estimated glomerular filtration rate (e-GFR) of < 60 ml/min/1.73 m^2^ [1]. Traditionally, the presence of albuminuria has been recognised as the hallmark of DKD (classical albuminuric DKD). However, recent studies have shown that, in addition to the classical albuminuric DKD phenotype, two new nonalbuminuric phenotypes of DKD exist, i.e., nonalbuminuric DKD and progressive renal decline, suggesting that progression of DKD can also occur through a non-albuminuric pathway [2, 3].

An increasing burden of DKD has been reported in adult patients with type 2 diabetes in sub-Saharan Africa (SSA) [4–6]. While there is reasonable data on the prevalence of DKD in African patients with long-standing diabetes, data on its burden and predictors in adult patients with newly diagnosed diabetes remains limited.

This study aimed to investigate the prevalence, phenotypes, and predictors of DKD in an adult Ugandan population with newly diagnosed diabetes.

## Methods

### Study settings, duration, and participants

The study participants were recruited from adult diabetes outpatient clinics of seven tertiary public and private mission hospitals located in Central and Southwestern Uganda between February 2019 and October 2020.

Participants were patients aged ≥ 18 years with new-onset diabetes (< 3 months preceding the diagnosis of diabetes), either treatment naïve or any glucose-lowering therapy. Participants with fever, any acute illness, and pregnancy were excluded.

A total of 519 participants were recruited for the study.

### Socio-demographic, clinical, anthropometric, and metabolic characterisation

After providing written informed consent, a study questionnaire was used to collect the vital sociodemographic (age, sex, and smoking status) and clinical (self-reported history of co-existing hypertension) characteristics. Resting blood pressure and anthropometric measurements were then performed and the systolic and diastolic blood pressure, body mass index (BMI), waist circumference (WC), hip circumference (HC), and waist: hip circumference ratio (WHR) were recorded.

All participants had a fasting blood sample drawn for the measurement of fasting blood glucose (FBG), glycated haemoglobin (HbA1c), lipid profile, uric acid, a complete blood count (for estimation of the haemoglobin level), serum urea, and serum creatinine (for estimation of the e-GFR).

A spot mid-stream urine sample was also collected for the measurement of UACR. This was performed using the Siemens Healthcare Clinitek® microalbumin reagent test strips and a point-of-care Clinitek® status analyser. The Clinitek® status analyser also provided data on urine leucocytes, nitrites, glucose, blood, and pH.

All the above tests were performed at the Medical Research Council/Uganda Virus Research Institute and London School of Hygiene and Tropical Medicine Uganda Research Unit, Entebbe Uganda.

The e-GFR was determined using the Chronic Kidney Disease Epidemiology (CKD-EPI) formula [7]. The e-GFR and the UACR were classified according to the Kidney Disease: Improving Global Outcomes (KDIGO) 2020 Clinical Practice Guideline for Diabetes Management in Chronic Kidney Disease. The e-GFR was categorized as follows: G1: ≥90mL/min/1.73m^2^ (normal kidney function), G2: 60–89mL/min/1.73 m^2^ (mildly decreased), G3a: 45–59mL/min/1.73 m^2^ (mildly to moderately decreased), G3b: 30–44mL/min/1.73 m^2^ (moderately to severely decreased), G4: 15–29mL/min/1.73 m^2^ (severely decreased), and G5: <15mL/min/1.73m^2^ (kidney failure). Albuminuria categories based on the UACR were as follows: A1: <3 mg/mmol (normal to mildly increased), A2: 3–30 mg/mmol (moderately increased), and A3: >30 mg/mmol (severely increased) [1].

### Definition of diabetic kidney disease and its phenotypes

DKD was defined as a spot UACR ≥ 3 mg/mmol (A2 and A3) with or without a reduced e-GFR of < 60 ml/min/1.73m^2^ (G3a-G5). Albuminuric and non-albuminuric DKD was defined as a spot UACR ≥ 3 mg/mmol (A2 and A3) regardless of the e-GFR and an e-GFR < 60 ml/min/1.73m^2^ with UACR < 3 mg/mmol (G3a-G5 and A1). No DKD was defined as an e-GFR ≥ 90 ml/min/1.73m^2^ and UACR < 3 mg/mmol (G1 and A1) [1].

### Statistical analysis

To describe the characteristics of all study participants, we used proportions for the categorical variables and medians with inter-quartile range (IQR) for the continuous variables. Proportions were also used to express those with and without DKD, and the two DKD phenotypes (albuminuric and non-albuminuric DKD).

The differences in the socio-demographic, clinical, anthropometric, and metabolic characteristics of participants with and without DKD were analysed using the *x*^2^ test for categorical data and the Kruskal Wallis test for continuous data. Odds ratios (OR) and their corresponding 95% confidence intervals (CI) were estimated using logistic regression. Specific sociodemographic, clinical, and metabolic characteristics well-known to be associated with DKD were added to the logistic regression model to identify the independent predictors of DKD. All analyses were done using STATA statistical software version 15 College Station, TX: StataCorp LLC.

## Results

The characteristics of all study participants and those with and without DKD separately are shown in Table 1.

**Table 1:** Socio-demographic, clinical, anthropometric, and metabolic characteristics of all participants, and participants with and without DKD separately

Characteristics	All study participants (n = 519)	DKD present (n = 175, 33.7%)	No DKD (n = 344, 66.3%)	P value
**Socio-demographic and clinical**				
Age (years) ≥ 50 years*	48 (39–57) 242 (46.6)	50 (42–59) 94 (53.7)	47 (39–56) 148 (43)	0.36 0.02
Sex* Males Females	227 (43.7) 292 (56.3)	68 (38.9) 107 (61.1)	159 (46.2) 185 (53.8)	0.11
Smoking habits (current or quit) *	45 (8.7)	12 (6.9)	33 (9.6)	0.57
Self-reported hypertension comorbidity*	174 (33.7)	71 (40.6)	103 (30.1)	0.02
SBP (mmHg)	125 (115–136)	126 (116–142)	125 (115–133)	0.50
DBP (mmHg)	84 (77–91)	85 (78–92)	83 (76–90)	0.38
**Anthropometric**				
BMI (kg/m^2^)	27.4 (23.4–31.4)	26.5 (23-31.3)	27.6 (23.7–31.6)	0.51
BMI < 25 kg/m^2^ 25-29.9 kg/m^2^ ≥30 kg/m^2^	174 (33.7) 164 (31.8) 178 (34.5)	66 (37.9) 53 (30.5) 55 (31.6)	108 (31.6) 111 (32.5) 123 (35.9)	0.34
WC (cm)	96 (87–104)	96 (87-104.1)	96 (87.5–104)	0.60
HC (cm)	103.5 (95.5-111.8)	102.5 (94.5-110.5)	104 (96–112)	0.67
WHR	0.92 (0.88–0.96)	0.93 (0.89–0.97)	0.92 (0.87–0.96)	0.63
**Metabolic**				
Haemoglobin (g/dl)	14.3 (13.2–15.4)	14.2 (12.8–15.4)	14.3 (13.5–15.4)	0.27
Urea (mmol/l)	3.2 (2.5–4.1)	3.2 (2.5–4.3)	3.1 (2.4-4)	0.20
e-GFR (ml/min/1.73m^2^)	121.8 (105.4-133.9)	117.5 (97.9-132.3)	124.6 (109.5-134.6)	0.41
TC (mmol/l)	4 (3.2-5)	4.1 (3.2-5)	4 (3.2-5.0)	0.11
HDLC (mmol/l)	0.9 (0.7–1.2)	1 (0.8–1.3)	0.9 (0.7–1.2)	0.30
TGL (mmol/l)	1.4 (1-1.9)	1.4 (1-1.9)	1.3 (1-1.8)	0.36
LDLC (mmol/l)	2.6 (1.9–3.4)	2.6 (1.8–3.52)	2.6 (2-3.3)	0.53
Non-HDLC (mmol/l)	3 (2.3–3.8)	3.1 (2.3–3.9)	3 (2.3–3.8)	0.43
Uric acid (µmol/l)	270 (217-330.2)	272 (214–335)	268 (217–327)	0.68
HbA1c (%)	10.6 (7.8–12.5)	10.8 (8.6–12.5)	10.4 (7.5–12.6)	0.44
HbA1c (mmol/mol)	92 (62–114)	95 (70–113)	90 (59–114)	0.43
FBG (mmol/l)	8.6 (6.3–13.4)	9.5 (6.5–14)	8.1 (6.2–12.8)	0.42

The categorical and continuous variables are presented as percentages and median (interquartile ranges), respectively

The median (IQR) age, HbA1c, UACR, and e-GFR of the participants was 48 years (39–57), 10.6% (7.8–12.5) or 92 mmol/mol (62–114), 2.27 mg/mmol (1.14–3.41), and 121.8 ml/min/1.73m^2^ (105.4-133.9). A UACR of < 3 mg/mol (A1 category), 3–30 mg/mmol (A2 category), and > 30 mg/mmol (A3 category) were noted in 344 (66.3%) participants, 167 (32.2%) participants, and 8 (1.5%) participants, respectively. An e-GFR of ≥ 90 ml/min/1.73m^2^ (G1 category), 60–90 ml/min/1.73m^2^ (G2 category), 45–59 ml/min/1.73m^2^ (G3a category), 30–44 ml/min/1.73m^2^ (G3b category), and < 15 ml/min/1.73m^2^ (G5 category) was noted in 461 (88.8%) participants, 51 (9.8%) participants, 4 (0.8%) participants, 2 (0.4%) participants, and 1 (0.2%) participant, respectively. No participant had an e-GFR between 15 and 29 ml/min/1.73m^2^.

DKD was observed in 175 participants (33.7%) participants.

### Socio-demographic, clinical, and metabolic characterisation of participants with and without DKD

Compared with those without DKD, participants with DKD generally were more likely to be ≥ 50 years of age (53.7% vs. 43%, p = 0.02) and have co-existing hypertension at the time of diagnosis (40.6% vs. 30.1%, p = 0.02). No significant differences in sex, anthropometric (BMI, WC, and WHR), and metabolic (glycaemic indices-FBG and HbA1c, lipid profile, and uric acid concentrations) characteristics were noted between the two groups.

### Independent predictors of DKD

Table 2 shows the multivariate analysis to identify predictors of DKD.

**Table 2:** Multivariate analysis to identify predictors of diabetic kidney disease

Characteristic	AOR (95%CI)	P-value
Age at diagnosis (years)	1.01 (1.00-1.02)	0.19
Sex of participants	1.26 (0.89–1.75)	0.19
Self-reported hypertension comorbidity	1.76 (1.24–2.48)	0.002
BMI < 25 kg/m2 25-29.9 kg/m2 ≥ 30 kg/m2	1 0.77 (0.52–1.15) 0.61 (0.41–0.91)	0.20 0.02

On multivariate analysis, self-reported hypertension comorbidity (OR 1.76 95% CI 1.24–2.48, p = 0.002) and BMI ≥ 30 kg/m^2^ (OR 0.61 95% CI 0.41–0.91, p = 0.02) were noted to be independent predictors of DKD.

## Discussion

In this study population, we report that DKD was relatively common, with the majority of participants presenting with the albuminuric phenotype. We also documented that pre-existing hypertension increased the odds of DKD while obesity reduced the risk.

A wide variation in the prevalence of DKD has been reported across African populations [4–6, 8, 9]. In one systematic review and meta-analysis of 21 medium- and high-quality studies performed in SSA to document the epidemiology of chronic kidney disease, the prevalence of DKD in the included studies was reported to range between 7.3% and 24%, with proteinuria being used as the main marker of the presence of DKD, in 96% of the studies [6]. In another systematic review of 32 studies (two population-based and 30 clinic-based) performed in Africa by Noubiap JJ et al., the prevalence of DKD varied from 11 to 83.7%, with about 63% of the studies diagnosing DKD based on urine protein measurement [4]. Another systematic review and meta-analysis that evaluated the burden of DKD and its association with hypertension in 27 studies performed in SSA reported a pooled prevalence of DKD of 35.3% [5], similar to what we documented in our study.

The reasons to explain these significant variations in the prevalence of DKD across the different African populations may be related to differences in diagnostic methods (urine protein and e-GFR measurement) and diagnostic thresholds used, populations studied (long-standing vs. newly diagnosed or type 1 vs. type 2 diabetes or combined), and study design (population-based vs. health facility-based or private vs. public health facilities).

In our study, pre-existing hypertension was documented to be associated with increased odds of having DKD. Hypertension is a well-recognised risk factor of DKD, regardless of phenotype, in African [5, 8, 10–12], white [13, 14], and Asian [15, 16] populations. The mechanisms to explain the association between hypertension and DKD are not well-understood but may be related to augmented sodium retention, pro-inflammatory cascade, renin-angiotensin-aldosterone, sympathetic nervous system, endothelial cell dysfunction, and oxidative stress, which ultimately result in glomerular damage [17, 18].

Obesity (BMI ≥ 30 kg/m^2^) was documented to reduce the odds of DKD in our study population, a finding conflicting with what has been observed in most studies investigating risk factors of DKD [19]. This underscores the heterogeneity in the pathogenesis of DKD across populations.

A similar protective effect of obesity towards having DKD was also reported in a retrospective study performed in rural South Africa in 253 adult patients with diabetes. Obesity and severe obesity defined as BMI > 27 kg/m^2^ and > 33 kg/m^2^ were present in 63% and 36.5% of the participants, respectively. On multivariate analysis, severe obesity was associated with reduced odds of having microalbuminuria (OR 0.27 95% CI 0.08–0.48, p = 0.002) [11]. A BMI > 25 kg/m^2^ was also noted to be associated with a reduced likelihood of chronic kidney disease, regardless of cause, in an urban and peri-urban community-based study performed in Uganda [20].

The reasons explaining the increased risk of DKD in individuals with normal or low BMI as reported in our study have not been investigated in great detail but could partly be explained by the developmental origins of health and disease (DOHaD) theory or developmental programming. Early-life environmental insults (in-utero or early childhood) like chronic infections, pre-eclampsia, and maternal malnutrition induce epigenetic changes that affect gene expression, development of critical body organs like the kidneys and pancreas, and function later in life resulting in several cardiometabolic conditions. Increased susceptibility to hypertension (a common predictor of kidney disease in African patients) and chronic kidney disease often develop due to an existing low nephron number or mass (Brenner hypothesis) which may be related to a pre-existing history of low BMI in the early stages of life [21]. This may explain why a high BMI, as reported in our study, is protective against DKD.

### Study limitations

We used a single-spot UACR measurement to determine participants with DKD. This could have led to an over-representation of the burden of DKD in this study population. We were also unable to screen for diabetic retinopathy, as recommended, to correlate the diagnosis of DKD, especially in patients with the albuminuric phenotype.

## Conclusion

DKD especially the albuminuric phenotype was relatively frequent in these adult Ugandan patients with newly diagnosed diabetes. A positive association was observed between hypertension comorbidity and DKD while obesity had an inverse association. Measurement of UACR as a measure of assessing for DKD should be encouraged in clinical care, especially in patients with co-existing hypertension and BMI < 30 kg/m^2^.

## Data Availability

The datasets used and/or analysed during the current study are available from the corresponding author on reasonable request.

## List of abbreviations

AOR: Adjusted odds ratio
BMI: Body mass index
CI: Confidence interval
CKD-EPI: Chronic Kidney Disease Epidemiology formula
DKD: Diabetic kidney disease
e-GFR: Estimated glomerular filtration rate
FBG: Fasting blood glucose
HbA1c: Glycated haemoglobin
HC: Hip circumference
KDIGO: Kidney Disease Improving Global Outcomes
SSA: Sub-Saharan Africa
UACR-: Urine albumin creatinine ratio
WC-: Waist circumference
WHR-waist: Hip circumference ratio
